# Novel Azetidine-Containing TZT-1027 Analogues as Antitumor Agents

**DOI:** 10.3390/md14050085

**Published:** 2016-04-28

**Authors:** Qi Yan, Yujie Wang, Wei Zhang, Yingxia Li

**Affiliations:** School of Pharmacy, Fudan University, Shanghai 201203, China; yanq12@fudan.edu.cn (Q.Y.); wyjbljy@163.com (Y.W.)

**Keywords:** TZT-1027, azetidine, conformation restriction, antiproliferative activity

## Abstract

A conformational restriction strategy was used to design and synthesize nine TZT-1027 analogues. 3-Aryl-azetidine moiety was used to replace phenylethyl group of TZT-1027 at the C-terminus. These analogues exhibited moderate to excellent antiproliferative activities, and the most potent compound **1a** showed IC_50_ values of 2.2 nM against A549 and 2.1 nM against HCT116 cell lines, respectively. However, **1a** could not achieve effective inhibition at all the dose levels in the A549 xenograft model (up to 5 mg/kg, injection, once a day), which is only 16%–35% inhibition at the end of the experiment.

## 1. Introduction

**Figure 1:**
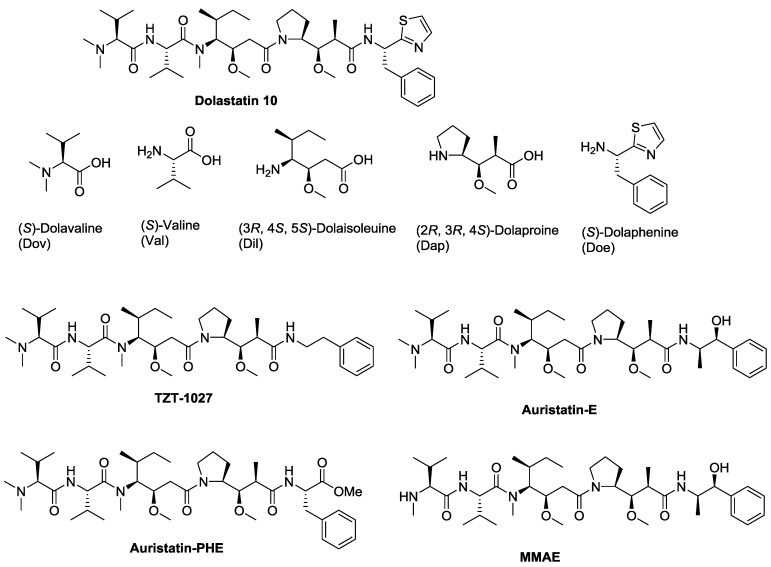
Structures of dolastatin 10 and its representative analogues.

Dolastatin 10 and its natural analogues are highly-cytotoxic peptides isolated from the sea hare *Dolabella auricularia* from the India Ocean [1]. These compounds have been demonstrated to be effective against a broad spectrum of cancer cells [2]. The extraordinary cytotoxicity is caused by their ability to inhibit microtubule assembly and tubulin-dependent guanosine triphosphate (GTP) hydrolysis, which result in cell cycle arrest and apoptosis [3]. A large number of synthetic analogues of dolastatin 10 have been reported [4,5,6]. Some of them, such as TZT-1027, auristatin E, and auristatin PHE were advanced into clinical trials (Figure 1). However, significant side effects were observed in clinical trials at dose levels that were not sufficient to attain clinical efficacy [7,8]. MMAE, a monomethyl analog of Auristatin-E, was conjugated to monoclonal antibodies, leading to the discovery of the FDA approved ADC brentuximab vedotin (ADCETRIS) for the treatment of relapsed Hodgkin lymphoma and systemic anaplastic large cell lymphoma [9].

**Figure 2:**
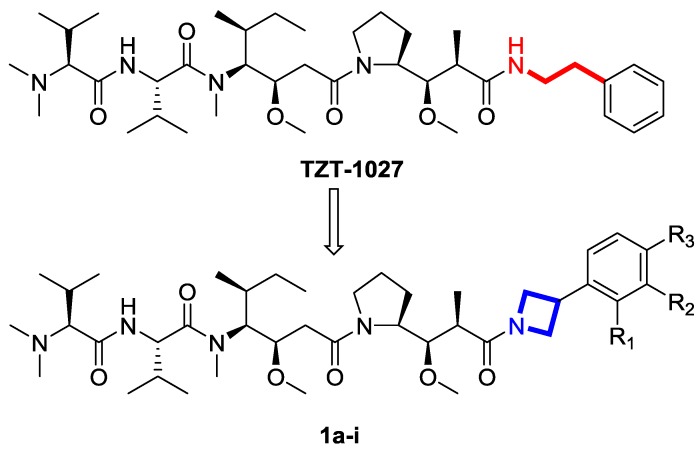
Designed target compounds.

Conformational study of dolastatin 10 analogues bound to tubulin revealed a compact structure that folded around the central Val-Dil bond in its *cis* form, whereas the flexible C-terminus does not interact with any amino acid residue directly, indicating that its main role might be arranging the molecule’s overall orientation [10,11]. Here we introduced azetidine moiety into C-terminus of TZT-1027 to explore the effect of conformational restriction on potency (Figure 2) [12]. Thus, nine conformational restricted analogues were synthesized and evaluated for inhibitory effects.

## 2. Results and Discussion

### 2.1. Chemistry

**Scheme 1:**
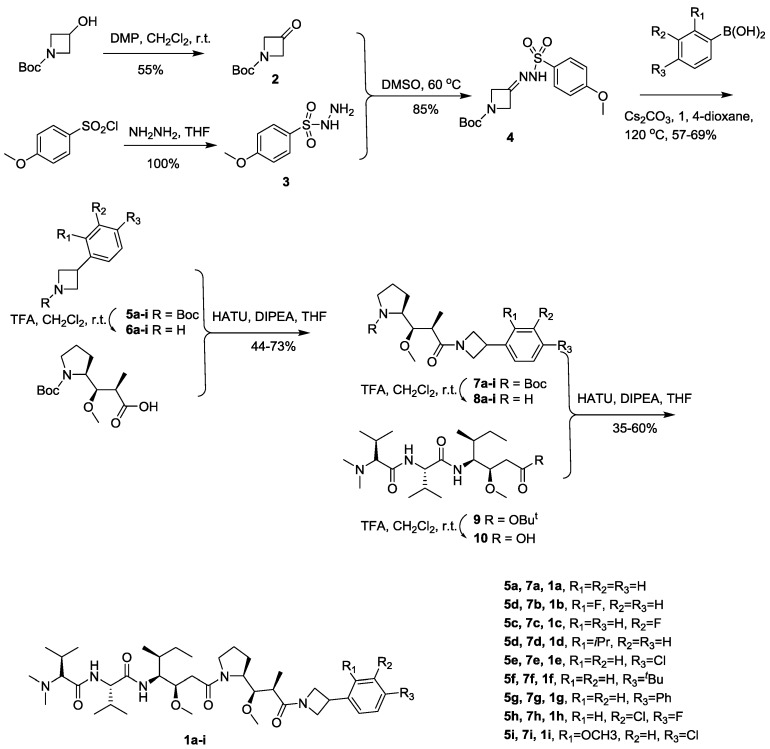
Synthetic route of target compounds.

The synthetic route is outlined in Scheme 1. 3-Aryl-azetidines **5a**–**i** were prepared according to known procedure [13]. Removal of the Boc group with trifluoroacetic acid (TFA) yielded the TFA salts **6a**–**i**, which were coupled with *N*-Boc-(2*R*, 3*R*, 4*S*)-dolaproine (Dap) in the presence of HATU to give compounds **7a**–**i**. Removal of the Boc group with TFA in **7a**–**i** yielded the TFA salts **8a**–**i**, which were coupled with Dov-Val-Dil·TFA (9) in the presence of HATU to provide the title compounds [5].

### 2.2. In Vitro Antiproliferative Assay

As shown in Table 1, these analogues demonstrated moderate to excellent antiproliferative activities. Among them, compound **1a** was the most potent with IC_50_ values of 2.2 nM against A549 cell lines and 2.1 nM against HCT116 cell lines. Structure-activity relationship could not be well illustrated due to a limited set of compounds. Basically, different substitutions on the phenyl group such as *ortho*-fluor (**1b**), *meta*-fluor (**1c**), *para*-chloro (**1e**), *para*-*tert*-butyl (**1f**), and *para*-phenyl (**1g**) could not improve the antiproliferative activities. These compounds resulted in about 20–30-fold loss of potency against A549 cell lines. When a bulky isopropyl group was introduced to the *ortho*-position of phenyl group, inhibitory activity was reduced about 60-folds (**1d**). All the target compounds showed weaker activity than TZT1027, indicating that conformational restriction at the C-terminus may not be beneficial to the activity. Membrane permeability can be a limiting factor for potency. The permeability data of synthesized compounds were not measured but we hypothesized that different substitutes of C-terminus could influence permeability, hence the antiproliferative activities. In addition, all the compounds showed better activity in HCT116 cell lines over A549 cell lines, demonstrating a cell selectivity. This is because HCT116 cells demonstrated a rapid proliferation rate than A549 cells and it is known that cytotoxic cancer drugs are believed to gain selectivity by targeting cells that proliferate rapidly.

### 2.3. Inhibitory Activity of Compound ***1a*** in A549 Xenograft Model

**Figure 3:**
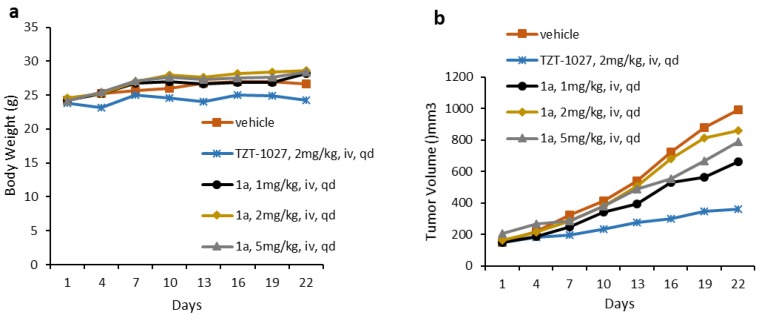
Antitumor activity of **1a** in A549 xenograft mice at different dosages. (**a**) Body weight and (**b**) tumor volume were measured on the indicated days after treated with vehicle or **1a** once a day.

Further *in vivo* antitumor activities of **1a** was evaluated in A549 xenograft models in mice via tail vein intravenous injection for 22 days. It is reported that a dose of 4 mg/kg of TZT-1027 seemed to be toxic [14,15]. Considering of that, the maximum dose of **1a** was chosen as 5 mg/kg. After given **1a** at 1 mg/kg/day, 2 mg/kg/day, and 5 mg/kg/day dosages, no overt toxicity and weight-loss were observed. However, compound **1a** could not achieve effective inhibition at all the dose levels (Figure 3b). TZT-1027 (2 mg/kg/day) inhibited tumor growth by 61% over the 22-day administration schedule, however **1a** only inhibited tumor growth by 16%–35% at difference dose (Supplementary Materials, Tables S1–S3). No time- and dosage-dependent inhibition were observed. Higher dosage of **1a** was not explored due to its poor solubility (Supplementary Materials, Table S4). Pharmacokinetic (PK) study was not conducted because in a mouse liver microsomes metabolic stability study, compound **1a** demonstrated a T_1/2_ of less than 2 min (Supplementary Materials, Table S5). The synthesis of analogues suitable for formulation is of considerable interest and this work will be reported in due course.

## 3. Experimental Section

### 3.1. Chemistry

#### 3.1.1. General

All starting materials, reagents, and solvents were commercially available. All reactions were monitored by thin-layer chromatography on silica gel plates (GF-254) and visualized with UV light. All the melting points were determined on a micromelting-point apparatus and thermometer was uncorrected. ^1^H-NMR spectra and ^13^C-NMR were recorded in acetone-*d_6_* or CDCl_3_ on a 400 or 600 Bruker NMR spectrometer with tetramethylsilane (TMS) as an internal reference. All chemical shifts are reported in parts per million (ppm). High-resolution exact mass measurements were performed using electrospray ionization (positive mode) on a quadrupole time-of-flight (QTOF) mass spectrometer (Maxis Q-TOF, Bruker Inc., Billerica, MA, USA).

#### 3.1.2. General Synthesis for 3-Aryl-Azetidines **5a**–**i**

To a solution of sulfonyl chloride (1.0 equiv) in THF (0.2 M) at 0 °C was added hydrazine hydrate (2.5 equiv) dropwise. The reaction mixture was stirred at 0 °C until complete conversion was observed by thin-layer chromatography. The mixture was diluted with EtOAc, washed with brine, dried over Na_2_SO_4_ and solvents removed *in vacuo* to give sulfonylhydrazides. To a solution of sulfonylhydrazones (1.0 equiv) in MeOH (0.5 M) was added ketone (1.0 equiv). The reaction mixture was stirred at room temperature until complete conversion was observed by TLC. Solvents were removed *in vacuo* to give sulfonylhydrazones. Sulfonylhydrazone (0.5 mmol, 1.0 equiv), boronic acid (0.75 mmol, 1.5 equiv), and cesium carbonate (0.75 mmol, 1.5 equiv) were placed in an oven-dried tube *in vacuo* for 30 min. The tube was backfilled with argon followed by the addition of dry degassed 1,4-dioxane (2 mL, 0.25 M). This tube was sealed and heated to 110 °C for 18 h before being cooled to room temperature, quenched with NaHCO_3_ (2 mL of a saturated aqueous solution), and extracted with CH_2_Cl_2_ (3 × 5 mL). The organic phase was dried over MgSO_4_, and solvents were removed *in vacuo* to give a residue, which was purified by flash column chromatography (10%−30% EtOAc/hexane) to give the title compounds.

*tert*-Butyl 3-phenylazetidine-1-carboxylate (**5a**). Colorless oil; yield 57%; ^1^H-NMR (400 MHz, CDCl_3_) δ 7.39–7.29 (m, 4H), 7.27–7.23 (m, 1H), 4.33 (t, *J* = 8.6 Hz, 2H), 3.98 (t, *J* = 8.6 Hz, 2H), 3.73 (tt, *J* = 8.6, 6.0 Hz, 1H), 1.47 (s, 3H); ^13^C-NMR (150 MHz, CDCl_3_) δ 156.5, 142.3, 128.8, 127.0, 126.8, 79.6, 33.6, 28.5; HRMS (ESI) calcd for C_14_H_19_NO_2_Na: 256.1308, found: 256.1307.

*tert*-Butyl 3-(2-fluorophenyl)azetidine-1-carboxylate (**5b**). Colorless oil, yield 69%; ^1^H-NMR (400 MHz, CDCl_3_) δ 7.31 (d, *J* = 7.8 Hz, 1H), 7.08 (d, *J* = 7.7 Hz, 1H), 7.02 (d, *J* = 9.9 Hz, 1H), 6.96 (t, *J* = 8.3 Hz, 1H), 4.33 (t, *J* = 8.7 Hz, 2H), 3.99–3.92 (m, 2H), 3.72–3.70 (m, 1H), 1.47 (s, 9H); ^13^C-NMR (150 MHz, CDCl_3_) δ 161.7, 160.1, 156.5, 128.8, 128.7, 128.6, 128.6, 128.0, 127.9, 124.4, 115.6, 115.4, 79.6, 29.8, 28.5, 27.6, 27.5; HRMS (ESI) calcd for C_14_H_18_NO_2_FNa: 274.1214, found: 256.1215.

*tert*-Butyl 3-(3-fluorophenyl)azetidine-1-carboxylate (**5c**). Colorless oil, yield 62%; ^1^H-NMR (400 MHz, CDCl_3_) δ 7.31 (td, *J* = 7.9, 6.1 Hz, 1H), 7.08 (d, *J* = 7.7 Hz, 1H), 7.03 (dt, *J* = 10.0, 2.1 Hz, 1H), 6.96 (td, *J* = 8.6, 2.9 Hz, 1H), 4.33 (t, *J* = 8.7 Hz, 2H), 3.96 (dd, *J* = 8.6, 5.9 Hz, 2H), 3.77–3.68 (m, 1H), 1.42 (s, 9H); ^13^C-NMR (150 MHz, CDCl_3_) δ 163.2, 161.6, 155.7, 144.1, 144.1, 129.7, 129.6, 129.5, 121.7, 121.7, 113.3, 113.2, 113.1, 79.1, 76.5, 76.3, 76.1, 32.6, 27.7, 27.6; HRMS (ESI) calcd for C_14_H_18_NO_2_FNa: 274.1214, found: 256.1215.

*tert*-Butyl 3-(2-isopropylphenyl)azetidine-1-carboxylate (**5d**). Colorless oil, yield 57%; ^1^H-NMR (400 MHz, CDCl_3_) δ 7.45–7.39 (m, 1H), 7.31–7.20 (m, 3H), 4.31 (t, *J* = 8.0 Hz, 2H), 4.13–4.05 (m, 1H), 4.03 (m, 2H), 1.46 (s, 10H), 1.21 (s, 3H), 1.19 (s, 3H); ^13^C-NMR (150 MHz, CDCl_3_) δ 156.6, 146.6, 138.3, 127.2, 126.3, 125.7, 125.4, 79.6, 29.8, 29.1, 28.5, 23.9; HRMS (ESI) calcd for C_17_H_25_NO_2_Na: 298.1778, found: 258.1777.

*tert*-Butyl 3-(4-chlorophenyl)azetidine-1-carboxylate (**5e**). Colorless oil, yield 50%; ^1^H-NMR (400 MHz, CDCl_3_) δ 7.30 (d, *J* = 8.1 Hz, 2H), 7.22 (d, *J* = 8.1 Hz, 2H), 4.31 (t, *J* = 8.7 Hz, 2H), 3.91 (dd, *J* = 8.6, 5.8 Hz, 2H), 3.72–3.63 (m, 1H), 1.46 (s, 9H); ^13^C-NMR (150 MHz, CDCl_3_) δ 161.0, 159.3, 155.7, 127.9, 127.8, 127.2, 127.2, 123.7, 123.6, 114.8, 114.7, 78.9, 27.7; HRMS (ESI) calcd for C_14_H_18_ClNO_2_Na: 290.0918, found: 290.0916.

*tert*-Butyl 3-(4-(tert-butyl)phenyl)azetidine-1-carboxylate (**5f**). Colorless oil, yield 53%; ^1^H-NMR (400 MHz,CDCl_3_) δ 7.38 (d, *J* = 8.3 Hz, 2H), 7.25 (d, *J* = 8.1 Hz, 2H), 4.31 (t, *J* = 8.6 Hz, 2H), 4.02–3.93 (m, 2H), 3.71–3.69 (m, 1H), 1.47 (s, 9H), 1.32 (s, 9H); ^13^C-NMR (150 MHz, CDCl_3_) δ 155.8, 149.2, 138.5, 125.8, 124.9, 78.8, 33.8, 32.4, 30.7, 27.8; HRMS (ESI) calcd for C_18_H_27_NO_2_Na: 312.1934, found: 312.1936.

*tert*-Butyl 3-([1,1′-biphenyl]-4-yl)azetidine-1-carboxylate (**5g**). Colorless oil, yield 66%; ^1^H-NMR (400 MHz, CDCl_3_) δ 7.58 (d, *J* = 7.8 Hz, 4H), 7.44 (t, *J* = 7.5 Hz, 2H), 7.39–7.32 (m, 3H), 4.35 (t, *J* = 8.6 Hz, 2H), 4.08–3.97 (m, 2H), 3.78–3.75 (m, 1H), 1.48 (s, 9H); ^13^C-NMR (150 MHz, CDCl_3_) δ 155.8, 140.6, 140.0, 139.3, 128.1, 128.1, 126.8, 126.6, 126.5, 126.4, 78.9, 27.8; HRMS (ESI) calcd for C_20_H_23_NO_2_Na: 332.1621, found: 332.1623.

*tert*-Butyl 3-(3-chloro-4-fluorophenyl)azetidine-1-carboxylate (**5h**). Colorless oil, yield 55%; ^1^H-NMR (400 MHz, CDCl_3_) δ 7.39–7.34 (m, 1H), 7.21–7.15 (m, 1H), 7.12 (t, *J* = 7.8 Hz, 1H), 4.33 (t, *J* = 8.7 Hz, 2H), 3.95–3.87 (m, 2H), 3.73–3.63 (m, 1H), 1.47 (s, 9H); ^13^C-NMR (150 MHz, CDCl_3_) δ 156.3, 139.3, 129.0, 126.5, 126.45, 121.2, 117.2, 116.8, 116.7, 114.7, 79.8, 56.5, 32.7, 28.4; HRMS (ESI) calcd for C_14_H_17_ClFNO_2_Na: 308.0830, found: 308.0829.

*tert*-Butyl 3-(4-chloro-2-methoxyphenyl)azetidine-1-carboxylate (**5i**). Colorless oil, yield 62%; ^1^H-NMR (400 MHz, CDCl_3_) δ 6.94 (dd, *J* = 8.1, 1.8 Hz, 1H), 6.83 (d, *J* = 1.7 Hz, 1H), 4.25 (t, *J* = 8.4 Hz, 2H), 4.04–3.86 (m, 3H), 3.80 (s, 3H), 1.45 (s, 9H); ^13^C-NMR (150 MHz, CDCl_3_) δ 157.2, 155.9, 132.6, 127.6, 127.1, 119.8, 110.4, 78.7, 54.9, 27.9, 27.8; HRMS (ESI) calcd for C_15_H_20_ClNO_3_Na: 320.1024, found: 320.1024.

#### 3.1.3. General Synthesis for **7a**–**i**

Compounds **5a**–**i** (1 equiv.) were dissolved in 2 mL CH_2_Cl_2_/TFA (1:1, *v*/*v*) at 0 °C, and the mixture was stirred for 1 h at room temperature. The reaction was then concentrated in vacuum, followed by azeotroping with dichloromethane three times to obtain the trifluoroacetate salts. To a stirring solution of the Dap in dry dichloromethane at 0 °C were sequentially added HATU (1.5 equiv.). After 10 min, the previously prepared trifluoroacetate salts dissolved in dichloromethane was added to reaction mixture followed by the addition of DIPEA (3 equiv.). After stirring for 12 h at room temperature, the reaction mixture was diluted with EtOAc/CH_2_Cl_2_, washed with 1M HCl, saturated NaHCO_3_ solution, water and brine, dried, filtered and concentrated in vacuo. Purification by silica gel column chromatography (EtOAc/Petroleum ether, 1/1) afforded compounds **7a**–**i**.

*tert*-Butyl (*S*)-2-((1*R*,2*R*)-1-methoxy-2-methyl-3-oxo-3-(3-phenylazetidin-1-yl)propyl)pyrrolidine-1-carboxylate (**7a**). Colorless oil, yield 57%; ^1^H-NMR (400 MHz, CDCl_3_) δ 7.39–7.34 (m, 2H), 7.30–7.29 (m, 3H), 4.58–4.51 (m, 1H), 4.45–4.34 (m, 1H), 4.20–4.01 (m, 2H), 3.91–3.87 (m, 1H), 3.78–3.76 (m, 2H), 3.46 (s, 3H), 3.29–3.23 (m, 1H), 2.41–2.37 (m, 1H), 2.01–1.93 (m, 2H), 1.85–1.72 (m, 1H), 4.52 (d, *J* = 10.6 Hz, 3H), 1.25 (s, 9H); ^13^C-NMR (150 MHz, CDCl_3_) δ 148.9, 139.4, 135.0, 128.5, 128.1, 127.7, 124.8, 124.5, 120.0, 53.5, 50.9, 41.9, 41.5, 28.1, 25.8, 19.5, 18.7, 12.1, 10.8. HRMS (ESI) calcd for C_23_H_35_N_2_O_4_: 403.2591, found: 403.2597; ${\left[\alpha \right]}_{D}^{25}$-35.000 (CHCl_3_, *c* = 0.50).

*tert*-Butyl (*S*)-2-((1*R*,2*R*)-3-(3-(2-fluorophenyl)azetidin-1-yl)-1-methoxy-2-methyl-3-oxopropyl)pyrrolidine-1-carboxylate (**7b**). Colorless oil, yield 60%; ^1^H-NMR (400 MHz, CDCl_3_) δ 7.27–7.10 (m, 1H), 6.96–6.94 (m, 1H), 6.83 (d, *J* = 1.7 Hz, 1H), 4.50–4.43 (m, 1H), 4.35–4.31 (m, 1H), 4.27–4.24 (m, 1H), 4.19–4.08 (m, 2H), 3.97–3.82 (m, 3H), 3.81 (s, 3H), 3.78–3.74 (m, 1H), 3.26–3.22 (m,1H), 1.95 (s, 3H), 1.92–1.68 (m, 4H), 1.52 (s, 3H), 1.44 (d, *J* = 7.8 Hz, 3H); ^13^C-NMR (150 MHz, CDCl_3_) δ 174.4, 154.6, 129.0, 128.1, 128.0, 124.5, 115.8, 84.2, 84.1, 82.1, 79.9, 79.1, 61.2, 60.8, 59.5, 59.3, 58.9, 56.4, 53.5, 53.3, 47.0, 46.7, 39.2, 38.4, 28.7, 28.6, 27.8, 27.4, 26.2, 26.1, 25.7, 24.7, 24.6, 24.3, 14.7, 14.6, 13.8; HRMS (ESI) calcd for C_23_H_33_N2O_4_FNa: 443.2317, found: 443.2318; ${\left[\alpha \right]}_{D}^{25}$-42.000 (CHCl_3_, *c* = 0.50).

*tert*-Butyl (*S*)-2-((1*R*,2*R*)-3-(3-(3-fluorophenyl)azetidin-1-yl)-1-methoxy-2-methyl-3-oxopropyl)pyrrolidine-1-carboxylate (**7c**). Colorless oil, yield 55%; ^1^H-NMR (400 MHz, CDCl_3_) 7.31–7.27 (m, 1H), 7.07 (d, *J* = 7.4 Hz, 1H), 7.02–6.97 (m, 2H), 4.64–4.49 (m, 2H), 4.40–4.35 (m, 1H), 4.26–4.03 (m, 1H), 4.02–3.96 (m, 1H), 3.94–3.84 (m, 1H), 3.83–3.74 (m, 1H), 3.64–3.50 (m, 1H), 3.45 (s, 3H), 3.30–3.21 (m, 2H), 1.99–1.91 (m, 3H), 1.89–1.72 (m, 2H), [1.52 (s), 1.47 (s), 1.44 (s), total 9H], 1.24 (d, *J* = 6.9 Hz, 3H); ^13^C-NMR (150 MHz, CDCl_3_) δ 163.9, 163.9, 162.3, 162.2, 154.5, 130.4, 122.3, 114.1, 113.6, 113.5, 84.3, 84.1, 82.6, 82.3, 79.8, 79.1, 61.0, 60.7, 60.4, 58.7, 57.4, 57.4, 54.8, 54.6, 46.9, 46.8, 38.5, 32.8, 28.6, 26.2, 24.1, 14.5, 14.2; HRMS (ESI) calcd for C_23_H_33_N_2_O_4_FNa: 443.2317, found: 443.2321; ${\left[\alpha \right]}_{D}^{25}$-66.200 (CHCl_3_, *c* = 0.50).

*tert*-Butyl (*S*)-2-((1*R*,2*R*)-3-(3-(2-isopropylphenyl)azetidin-1-yl)-1-methoxy-2-methyl-3-oxopropyl)pyrrolidine-1-carboxylate (**7d**). Colorless oil, yield 65%; ^1^H-NMR (400 MHz, CDCl_3_) 7.37–7.35 (m, 1H), 7.28–7.24 (m, 3H), 4.56–4.51 (m, 1H), 4.43–4.34 (m, 1H), 4.22–4.07 (m, 3H), 3.88–3.77 (m, 3H), 3.57–3.55 (m, 1H), [3.45 (s) and 3.44 (s), total 3H], 3.26–3.23 (m, 1H), 3.01–2.93 (m, 1H), 2.50–2.39 (m, 1H), 1.95–1.72 (m, 6H), [1.51 (s), 1.47 (s) and 1.43 (s), total 9H], 1.25–1.19 (m, 9H); ^13^C-NMR (150 MHz, CDCl_3_) δ 173.6, 173.3, 153.9, 145.9, 137.2, 126.8, 125.6, 124.8, 124.6, 124.5, 83.6, 83.4, 81.9, 81.8, 79.2, 78.4, 60.5, 58.2, 58.1, 56.3, 56.2, 53.6, 53.4, 46.3, 46.0, 38.5, 37.9, 29.0, 28.5, 28.4, 28.0, 27.9, 25.5, 23.5, 23.2, 23.1, 13.9; HRMS (ESI) calcd for C_26_H_41_N_2_O_4_: 445.3061, found: 445.3065; ${\left[\alpha \right]}_{D}^{25}$-46.200 (CHCl_3_, *c* = 0.50).

*tert*-Butyl (*S*)-2-((1*R*,2*R*)-3-(3-(4-chlorophenyl)azetidin-1-yl)-1-methoxy-2-methyl-3-oxopropyl)pyrrolidine-1-carboxylate (**7e**). Colorless oil, yield 45%; ^1^H-NMR (400 MHz, CDCl_3_) 7.33–7.32 (m, 2H), 7.24–7.22 (m, 2H), 4.58–4.36 (m, 2H), 4.17–3.75 (m, 5H), 3.60–3.55 (m, 1H), [3.45 (s) and 3.44 (s), total 3H], 3.30–3.24 (m, 1H), 2.47–2.40 (m, 1H), 1.95–1.74 (m, 6H), [1.52 (s), 1.47 (s) and 1.44 (s), total 9H], 1.23 (d, *J* = 7.4 Hz, 3H); ^13^C-NMR (150 MHz, CDCl_3_) δ 173.9, 173.6, 173.4, 153.8, 139.6, 139.4, 132.4, 128.3, 127.3, 83.6, 83.4, 81.9, 81.7, 79.2, 78.4, 60.4, 60.1, 58.1, 56.9, 54.2, 54.1, 46.3, 46.0, 37.9, 37.8, 31.9, 29.0, 28.0, 25.6, 23.5; HRMS (ESI) calcd for C_23_H_33_ClN_2_O_4_Na: 459.2021, found: 459.2024; ${\left[\alpha \right]}_{D}^{25}$-46.600 (CHCl_3_, *c* = 0.50).

*tert*-Butyl (*S*)-2-((1*R*,2*R*)-3-(3-(4-(tert-butyl)phenyl)azetidin-1-yl)-1-methoxy-2-methyl-3-oxopropyl)pyrrolidine-1-carboxylate (**7f**). Colorless oil, yield 73%; ^1^H-NMR (400 MHz, CDCl_3_) δ 7.39 (d, *J* = 6.8 Hz, 2H), 7.24 (d, *J* = 8.1 Hz, 2H), 4.56–4.51 (m, 1H), 4.43–4.32 (m, 1H), 4.17–4.01 (m, 3H), 3.89–3.74 (m, 3H), 3.60–3.55 (m, 1H), [3.46 (s) and 3.45 (s), total 3H], 3.30–3.25 (m, 1H), 2.47–2.39 (m, 2H), 1.98–1.74 (m, 6H), [1.52 (s), 1.47 (s) and 1.45 (s), total 9H], [1.32 (s) and 1.31 (s), total 9H], 1.24 (d, *J* = 7.4 Hz, 3H); ^13^C-NMR (150 MHz, CDCl_3_) δ 173.9, 173.7, 173.6, 173.3, 153.8, 149.6, 149.5, 138.1, 137.9, 125.7, 125.1, 83.6, 83.4, 81.9, 81.8, 79.2, 78.4, 60.50, 58.1, 57.1, 54.4, 54.2, 46.0, 37.8, 33.8, 32.1, 31.9, 30.6, 28.0, 27.9, 25.6, 25.5, 23.6, 13.9; HRMS (ESI) calcd for C_27_H_43_N_2_O_4_: 4059.3217, found: 459.3220; ${\left[\alpha \right]}_{D}^{25}$-73.200 (CHCl_3_, *c* = 1).

*tert*-Butyl (*S*)-2-((1*R*,2*R*)-3-(3-([1,1′-biphenyl]-4-yl)azetidin-1-yl)-1-methoxy-2-methyl-3-oxopropyl)pyrrolidine-1-carboxylate (**7g**). Colorless oil, yield 45%; ^1^H-NMR (400 MHz, CDCl_3_) δ 7.59 (d, *J* = 7.1 Hz, 4H), 7.45 (t, *J* = 7.6 Hz, 3H), 7.46–7.33 (m, 3H), 4.60–4.53 (m, 1H), 4.47–4.36 (m, 1H), 4.25–4.04 (m, 3H), 3.89–3.79 (m, 3H), 3.60–3.55 (m, 1H), 3.46 (s, 3H), 3.28–3.25 (m, 1H), 2.53–2.41 (m, 1H), 1.97–1.75 (m, 5H), [1.52 (s), 1.48 (s) and 1.44 (s), total 9H], 1.25 (d, *J* = 7.4 Hz, 3H); ^13^C-NMR (150 MHz, CDCl_3_) δ 173.6, 173.4, 153.8, 139.9, 139.6, 128.2, 126.9, 126.7, 126.3, 83.6, 83.4, 81.9, 81.8, 79.2, 78.5, 60.5, 58.1, 57.1, 57.0, 54.3, 46.0, 37.8, 32.2, 32.1, 28.0, 27.9, 25.6, 23.5, 14.0. HRMS (ESI) calcd for C_29_H_38_N_2_O_4_: 479.2904, found: 4479.2910; ${\left[\alpha \right]}_{D}^{25}$-36.500 (CHCl_3_, *c* = 0.50).

*tert*-Butyl (*S*)-2-((1*R*,2*R*)-3-(3-(3-chloro-4-fluorophenyl)azetidin-1-yl)-1-methoxy-2-methyl-3-oxopropyl)pyrrolidine-1-carboxylate (**7h**). Colorless oil, yield 63%; ^1^H-NMR (400 MHz, acetone-*d_6_*) δ 7.58–7.54 (m, 1H), 7.39–7.34 (m, 2H), 4.57–4.45 (m, 1H), 4.24–4.02 (m, 4H), 3.88–3.59 (m, 5H), 3.31 (s, 3H), 3.15–3.09 (m, 1H), 1.82–1.64 (m, 6H), 1.39 (s, 9H), 0.06 (d, *J* = 7.4 Hz, 3H); ^13^C-NMR (150 MHz, acetone-*d_6_*) δ 173.1, 172.9, 156.7, 155.1, 153.1, 140.1, 139.7, 139.7, 129.4, 128.9, 128.7, 127.4, 127.3, 127.2, 119.4, 119.3, 119.2, 116.9, 116.8, 116.7, 83.7, 83.6, 81.8, 81.7, 78.4, 78.0, 60.3, 60.1, 58.6, 58.2, 56.8, 56.4, 54.2, 46.4, 46.2, 31.3, 28.0, 25.4, 24.9, 23.9, 23.4, 14.0; HRMS (ESI) calcd for C_23_H_32_ClFN_2_O_4_Na: 477.1927, found: 477.1927; ${\left[\alpha \right]}_{D}^{25}$-35.000 (CHCl_3_, *c* = 0.50).

*tert*-Butyl (*S*)-2-((1*R*,2*R*)-3-(3-(4-chloro-2-methoxyphenyl)azetidin-1-yl)-1-methoxy-2-methyl-3-oxopropyl)pyrrolidine-1-carboxylate (**7i**). Colorless oil, yield 44%; ^1^H-NMR (400 MHz, CDCl_3_) δ 7.12–7.069 (m, 1H), 6.95–6.92 (m, 1H), 6.85–6.84 (m, 1H), 4.48–4.43 (m, 1H), 4.33–4.05 (m, 3H), 3.96–3.81 (m, 2H), 3.80 (s, 3H), 3.77–3.73 (m, 1H), 3.56–3.54 (m, 1H), [3.44 (s) and 3.43 (s), total 3H], 1.95–1.51 (m, 5H), [1.50 (s), 1.46 (s) and 1.42 (s), total 9H], [1.22 (d, *J* = 6.9 Hz) and 1.18 (d, *J* = 6.9 Hz), total 3H]; ^13^C-NMR (150 MHz, CDCl_3_) δ 173.6, 157.3, 153.8, 127.3, 127.2, 119.9, 110.6, 83.4, 83.3, 81.8, 79.2, 78.4, 60.5, 58.2, 58.1, 55.2, 54.9, 54.9, 52.0, 51.9, 46.3, 46.0, 37.9, 29.0, 28.0, 27.9, 25.5, 23.5, 14.0, 13.8; HRMS (ESI) calcd for C_24_H_36_N_2_O_5_: 467.2307, found: 467.2311; ${\left[\alpha \right]}_{D}^{25}$-32.300 (CHCl_3_, *c* = 0.50).

#### 3.1.4. General Synthesis for **1a**–**i**

Commercially available tripeptide (1 equiv.) and **7a**–**i** (1 equiv.) was dissolved in CH_2_Cl_2_/TFA (1:1, *v*/*v*). After stirred for 2 h at room temperature, the solvent was removed in vacuum, followed by azeotroping with CH_2_Cl_2_ three times. Then the mixture was dissolved in dry CH_2_Cl_2_. DIPEA was added until reaction mixture was basic, followed by HATU (1.5 equiv.). After stirring for 12 h at room temperature, the reaction mixture was diluted with EtOAc, washed with 1M HCl, saturated NaHCO_3_ solution, water and brine, dried, filtered, and concentrated in vacuum. Purification by silica gel column chromatography (CH_2_Cl_2_/MeOH, 20/1) afforded the title compounds **1a**–**i**.

(*S*)-2-((*R*)-2-(Dimethylamino)-3-methylbutanamido)-*N*-((3*R*,4*S*,5*S*)-3-methoxy-1-((*S*)-2-((1*R*,2*R*)-1-methoxy-2-methyl-3-oxo-3-(3-phenylazetidin-1-yl)propyl)pyrrolidin-1-yl)-5-methyl-1-oxoheptan-4-yl)-*N*,3-dimethylbutanamide (**1a**). Colorless oil, yield 45%; ^1^H-NMR (400 MHz, CDCl_3_) 7.40–7.36 (m, 2H), 7.31–7.27 (m, 3H), 6.93 (d, *J* = 9.1 Hz, 1H), [4.87 (t, *J* = 7.2 Hz) and 4.79 (t, *J* = 7.8 Hz), total 1H], 4.66–4.59 (m, 1H), 4.54–4.46 (m, 1H), 4.45–4.40 (m, 1H), 4.37–4.32 (m, 1H), 4.28–4.23 (m, 1H), 4.21–3.77 (m, 11H), [3.43 (s) and 3.41 (s), total 3H], [3.36 (s) and 3.35 (s), total 3H], [3.34 (s) and 3.30 (s), total 3H], 3.14 (d, *J* = 6.2 Hz, 1H), 3.03–3.02 (m, 2H), 2.63–0.79 (m, 36H); ^13^C-NMR (150 MHz, CDCl_3_) 174.4, 147.1, 173.8, 173.7, 173.1, 171.2, 170.2, 169.9, 161.8, 161.7, 160.1, 129.2, 129.1, 129.0, 128.8, 128.4, 128.2, 128.1, 127.9, 127.7, 124.5, 124.4, 116.0, 115.8, 115.7, 115.6, 115.5, 86.4, 82.6, 82.4, 78.3, 77.8, 76.0, 61.9, 61.8, 60.5, 60.4, 59.4, 59.2, 59.0, 58.1, 57.9, 57.8, 56.5, 56.4, 56.2, 56.0, 55.8, 53.8, 53.6, 53.4, 53.2, 47.7, 47.5, 46.6, 46.5, 42.6, 39.4, 38.8, 37.7, 37.4, 35.8, 33.2, 33.1, 32.3, 31.8, 30.9, 28.4, 27.8, 27.7, 27.3, 26.2, 26.0, 25.7, 25.0, 24.9, 24.6, 23.6, 23.5, 20.1, 19.8, 19.5, 17.9, 17.8, 15.8, 15.4, 14.8, 14.6, 13.7, 13.5, 10.8, 10.7, 10.3; HRMS (ESI) calcd for C_40_H_68_N_5_O_6_: 714.5164, found: 714.5169; ${\left[\alpha \right]}_{D}^{25}$-50.200 (MeOH, *c* = 0.50).

(*S*)-2-((*R*)-2-(Dimethylamino)-3-methylbutanamido)-*N*-((3*R*,4*S*,5*S*)-1-((*S*)-2-((1*R*,2*R*)-3-(3-(2-fluorophenyl)azetidin-1-yl)-1-methoxy-2-methyl-3-oxopropyl)pyrrolidin-1-yl)-3-methoxy-5-methyl-1-oxoheptan-4-yl)-*N*,3-dimethylbutanamide (**1b**). Colorless oil, yield 40%; ^1^H-NMR (400 MHz, acetone-*d_6_*) δ 7.45–7.38 (m, 1H), 7.36–7.31 (m 1H), 7.23–7.19 (m, 1H), 7.16–7.11 (m, 1H), 4.80–4.63 (m, 3H), 4.45–4.33 (m, 2H), 4.15–3.93 (m, 4H), 3.68–3.55 (m, 2H), [3.42 (s) and 3.33 (s), total 3H], [3.30 (s) and 3.13 (s), total 3H], 2.75–2.05 (m, 10H), 1.20–0.79 (m, 24H); ^13^C-NMR (150 MHz, acetone-*d_6_*) δ 174.2, 174.2, 172.4, 170.1, 161.2, 159.6, 128.5, 128.4, 128.4, 128.0, 127.9, 127.5, 127.4, 127.3, 125.5, 124.0, 114.8, 114.7, 114.6, 81.4, 81.3, 78.7, 73.9, 60.2, 59.0, 58.9, 56.2, 55.2, 53.8, 52.9, 47.1, 47.0, 41.1, 38.2, 37.2, 36.0, 31.7 30.5, 29.6, 29.1, 27.1, 27.0, 26.6, 25.0, 24.2, 23.4, 18.5, 18.1, 17.6, 17.1, 16.9, 14.8, 13.3; HRMS (ESI) calcd for C_40_H_67_FN_5_O_6_: 732.5070, found: 732.5088; ${\left[\alpha \right]}_{D}^{25}$-35.800 (CHCl_3_, *c* = 0.50).

(*S*)-2-((*R*)-2-(Dimethylamino)-3-methylbutanamido)-*N*-((3*R*,4*S*,5*S*)-1-((*S*)-2-((1*R*,2*R*)-3-(3-(3-fluorophenyl)azetidin-1-yl)-1-methoxy-2-methyl-3-oxopropyl)pyrrolidin-1-yl)-3-methoxy-5-methyl-1-oxoheptan-4-yl)-*N*,3-dimethylbutanamide (**1c**). Colorless oil, yield 54%; ^1^H-NMR (400 MHz, CDCl_3_) δ 7.29–7.23 (m, 2H), 7.19–7.11 (m, 1H), 7.06–6.97 (m, 1H), 4.85–4.74 (m, 1H), 4.60–4.54 (m, 1H), 4.52–4.73 (m, 1H), 4.41–4.34 (m, 1H), 4.23–4.17 (m, 2H), 4.12–3.85 (m, 5H), 3.48–3.45 (m, 2H), [3.41 (s) and 3.40 (s), total 3H], [3.31 (s) and 3.29 (s), total 3H], 3.05 (s, 3H), 2.64–2.38 (m, 4H), 2.37–2.27 (m, 6H), 2.18–1.77 (m, 10H), 1.37–0.76 (m, 21H); ^13^C-NMR (150 MHz, CDCl_3_) δ 174.1, 174.0, 172.4, 170.6, 169.9, 169.6, 161.2, 159.6, 159.4, 128.7, 128.3, 128.3, 127.8, 127.2, 127.1, 126.9, 124.1, 123.8, 115.0, 114.9, 114.8, 114.7, 81.3, 79.0, 75.4, 68.9, 60.6, 59.0, 58.9, 58.4, 57.5, 56.8, 56.8, 56.0, 55.6, 53.1, 52.7, 52.6, 47.2, 42.0, 42.0, 38.5, 38.3, 36.2, 32.0, 31.1, 30.8, 30.2, 29.0, 28.6, 27.3, 26.9, 25.0, 24.3, 24.2, 23.8, 23.7, 19.4, 19.3, 19.1, 18.7, 17.6, 17.0, 15.1, 13.7, 13.6; HRMS (ESI) calcd for C_40_H_67_FN_5_O_6_: 732.5070, found: 732.5078; ${\left[\alpha \right]}_{D}^{25}$-35.200 (MeOH, *c* = 0.50).

(*S*)-2-((*R*)-2-(Dimethylamino)-3-methylbutanamido)-*N*-((3*R*,4*S*,5*S*)-1-((*S*)-2-((1*R*,2*R*)-3-(3-(2-isopropylphenyl)azetidin-1-yl)-1-methoxy-2-methyl-3-oxopropyl)pyrrolidin-1-yl)-3-methoxy-5-methyl-1-oxoheptan-4-yl)-*N*,3-dimethylbutanamide (**1d**). Colorless oil, yield 48%; ^1^H-NMR (400 MHz, CDCl_3_) δ 7.45–7.40 (m, 1H), 7.33–7.28 (m, 2H), 7.26–7.17 (m, 1H), 5.11–4.69 (m, 3H), 4.35–4.22 (m, 4H), 4.06–4.02 (m, 3H), 3.57–3.55 (m, 2H), [3.40 (s) and 3.38 (s), total 3H), 3.39–3.01 (m, 10H), 2.66–2.45 (m, 8H), 2.20–1.95 (m, 6H), 1.88–1.71 (m, 4H), 1.41–1.35 (m, 2H), 1.21–1.13 (m, 8H), 1.02–0.76 (m, 19H); 13C-NMR (150 MHz, CDCl3) δ 173.9, 173.6, 173.1, 173.0, 172.4, 169.7, 169.6, 169.0, 167.6, 167.5, 159.9, 159.7, 148.0, 145.9, 145.8, 139.2, 137.3, 134.3, 127.3, 126.5, 126.4, 126.4, 125.5, 125.5, 124.8, 124.7, 124.6, 124.6, 124.5, 124.5, 118.8, 117.5, 115.5, 81.56 81.4, 72.6, 72.5, 60.3, 59.8, 59.0, 58.9, 58.3, 56.7, 56.6, 56.3, 56.3, 55.9, 54.0, 53.7, 53.6, 53.4, 46.9, 45.4, 45.3, 40.4, 40.4, 38.3, 38.2, 38.0, 31.8, 29.8, 29.7, 29.0, 28.9, 28.8, 26.8, 26.8, 25.4, 25.3, 25.0, 24.1, 24.0, 23.8, 23.7, 22.8, 22.6, 18.5, 18.4, 18.1, 17.6, 17.5, 17.3, 17.3, 14.8, 13.5, 13.0, 12.7, 9.4; HRMS (ESI) calcd for C43H74N5O6: 778.5453, found: 778.5453; ${\left[\alpha \right]}_{D}^{25}$-40.200 (CHCl_3_, *c* = 1).

(*S*)-*N*-((3*R*,4*S*,5*S*)-1-((*S*)-2-((1*R*,2*R*)-3-(3-(4-Chlorophenyl)azetidin-1-yl)-1-methoxy-2-methyl-3-oxopropyl)pyrrolidin-1-yl)-3-methoxy-5-methyl-1-oxoheptan-4-yl)-2-((*R*)-2-(dimethylamino)-3-methylbutanamido)-*N*,3-dimethylbutanamide (**1e**). Colorless oil, yield 66%; ^1^H-NMR (400 MHz, CDCl_3_) δ 7.44–7.37 (m, 4H), 4.83–4.67 (m, 3H), 4.40–4.13 (m, 4H), 4.02–3.83 (m, 4H), 3.60–3.55 (m, 3H), [3.40 (s) and 3.37 (s), total 3H], [3.31 (s) and 3.26 (s), total 3H], 3.22–3.09 (m, 4H), 2.68–2.39 (m, 5H), 2.28 (s, 3H), 2.27 (s, 3H), 2.14–1.80 (m, 10H), 1.20–1.13 (m, 4H), 1.02–0.79 (m, 21H); ^13^C-NMR (150 MHz, CDCl_3_) δ 173.2, 172.9, 172.7, 169.7, 169.6, 168.8, 168.7, 141.1, 140.8, 140.6, 140.5, 131.5, 131.4, 128.1, 128.0, 127.9, 127.8, 85.7, 85.5, 81.8, 77.8, 77.6, 77.2, 74.3, 74.2, 60.3, 59.1, 58.6, 58.5, 58.2, 58.1, 56.6, 56.5, 54.2, 54.1, 54.0, 53.9, 53.2, 53.1, 46.5, 46.3, 45.6, 41.0, 38.5, 38.0, 37.9, 36.8, 36.5, 35.2, 31.9, 31.7, 29.9, 28.7, 28.6, 28.5, 28.3, 28.2, 28.1, 27.9, 26.6, 25.5, 25.4, 25.0, 24.9, 24.4, 24.0, 23.7, 22.8, 18.7, 18.5, 18.2, 17.8 17.4, 17.3, 14.8, 14.5, 13.6, 13.5, 12.4, 11.9, 9.5, 9.2; HRMS (ESI) calcd for C_40_H_67_ClN_5_O_6_: 748.4774, found: 748.4778; ${\left[\alpha \right]}_{D}^{25}$-34.500 (CHCl_3_, *c* = 0.50).

(*S*)-*N*-((3*R*,4*S*,5*S*)-1-((*S*)-2-((1*R*,2*R*)-3-(3-(4-(tert-Butyl)phenyl)azetidin-1-yl)-1-methoxy-2-methyl-3-oxopropyl)pyrrolidin-1-yl)-3-methoxy-5-methyl-1-oxoheptan-4-yl)-2-((*R*)-2-(dimethylamino)-3-methylbutanamido)-*N*,3-dimethylbutanamide (**1f**). Colorless oil, yield 35%; ^1^H-NMR (400 MHz, CDCl_3_) δ 7.44–7.35 (m, 2H), 7.26–7.15 (m, 2H), 4.86–4.69 (m, 3H), 4.64–4.51 (m, 2H), 4.22–4.01 (m, 2H), 4.01–3.76 (m, 4H), 3.75–3.66 (m, 4H), 3.55–3.47 (m, 5H), [3.42 (s) and 3.39 (s), total 3H], [3.29 (s) and 3.28 (s), total 3H], 3.24–3.11 (m, 3H), 3.03–2.62 (m, 1H), 2.59–2.27 (m, 4H), 2.21–1.69 (m, 3H), 1.47–1.15 (m, 10H), 1.05–0.71 (m, 18H); ^13^C-NMR (150 MHz, CDCl_3_) δ 174.0, 173.9, 172.5, 170.1, 170.0, 149.8, 149.7, 137.5, 137.2, 125.6, 125.1, 81.2, 81.0, 78.8, 60.7, 59.2, 59.0, 57.5, 57.2, 56.8, 54.8, 54.6, 47.6, 47.4, 42.8, 41.9, 41.8, 38.5, 38.4, 33.8, 31.9, 31.5, 31.3, 30.6, 30.2, 28.6, 26.9, 25.1, 24.3, 24.2, 23.8, 19.2, 19.0, 18.4, 18.4, 17.8, 17.0, 16.6, 15.1, 14.0, 13.8, 12.0, 10.0; HRMS (ESI) calcd for C_44_H_76_N_5_O_6_: 770.5790, found: 770.5809; ${\left[\alpha \right]}_{D}^{25}$-36.000 (CHCl_3_, *c* = 0.50).

(*S*)-*N*-((3*R*,4*S*,5*S*)-1-((*S*)-2-((1*R*,2*R*)-3-(3-([1,1′-Biphenyl]-4-yl)azetidin-1-yl)-1-methoxy-2-methyl-3-oxopropyl)pyrrolidin-1-yl)-3-methoxy-5-methyl-1-oxoheptan-4-yl)-2-((*R*)-2-(dimethylamino)-3-methylbutanamido)-*N*,3-dimethylbutanamide (**1g**). Colorless oil, yield 70%; ^1^H-NMR (400 MHz, CDCl_3_) δ 7.60 (d, J = 8.4 Hz, 4H), 7.47–7.44 (m, 2H), 7.40–7.34 (m, 3H), 4.89–4.79 (m, 3H), 4.68–4.65 (m, 1H), 4.58–4.47 (m, 1H), 4.45–4.39 (m, 1H), 4.23–3.85 (m, 10H), [3.45 (s) and 3.42 (s), total 3H], [3.37 (s) and 3.32 (s), total 3H], 3.16–3.03 (m, 4H), 2.80–2.02 (m, 10H), 1.26–0.82 (m, 23H); ^13^C-NMR (150 MHz, CDCl_3_) δ 173.8, 173.5, 169.6, 169.2, 139.8, 139.5, 128.1, 127.0, 126.9, 126.7, 126.5, 126.4, 126.3, 126.2, 81.9, 75.7,58.5, 58.4, 57.3, 57.2, 57.0, 56.9, 54.4, 53.1, 42.1, 38.2, 32.6, 32.5, 32.4, 32.2, 32.1, 31.2, 30.3, 29.0, 28.7, 27.0, 25.7, 25.1, 24.5, 24.4, 24.1, 23.0, 22.0, 19.4, 19.2, 18.9, 17.1, 15.2, 14.1, 13.4, 13.0, 10.1, 9.7; HRMS (ESI) calcd for C_46_H_72_N_5_O_6_: 790.5477, found: 790.5475; ${\left[\alpha \right]}_{D}^{25}$-20.900 (CHCl_3_, *c* = 1).

(S)-*N*-((3*R*,4*S*,5*S*)-1-((*S*)-2-((1*R*,2*R*)-3-(3-(3-Chloro-4-fluorophenyl)azetidin-1-yl)-1-methoxy-2-methyl-3-oxopropyl)pyrrolidin-1-yl)-3-methoxy-5-methyl-1-oxoheptan-4-yl)-2-((*R*)-2-(dimethylamino)-3-methylbutanamido)-*N*,3-dimethylbutanamide (**1h**). Colorless oil, yield 44%; ^1^H-NMR (400 MHz, CDCl_3_) δ 7.37–7.35 (m, 1H), 7.16–7.09 (m, 2H), 4.90–4.77 (m, 2H), 4.64–4.60 (m, 1H), 4.52–4.40 (m, 2H), 4.18–4.00 (m, 4H), 3.80–3.76 (m, 3H), [3.43 (s) and 3.41 (s), total 3H], [3.38 (s) and 3.36 (s), total 3H], 3.34–3.30 (m, 4H), 3.18–3.02 (m, 4H), 2.37–2.00 (m, 7H), 1.94–1.70 (m, 10H), 1.42–0.80 (m, 13H); ^13^C-NMR (150 MHz, CDCl_3_) δ 174.1, 174.0, 172.4, 170.6, 169.6, 161.2, 161.0, 159.6, 159.4, 128.3, 127.8, 127.1, 124.1, 123.8, 115.0, 114.9, 114.8, 114.7, 81.3, 79.0, 68.9, 60.6, 59.0, 58.9, 58.4, 57.5, 56.8, 56.0, 55.6, 53.1, 52.7, 52.6, 47.2, 42.0, 42.0, 38.5, 38.3, 36.2, 32.0, 31.1, 30.8, 30.2, 29.0, 28.6, 27.3, 26.9, 25.0, 24.3, 24.2, 23.8, 23.7, 19.4, 19.3, 19.1, 18.7, 17.6, 17.0, 15.1, 13.7, 13.6, 10.0; HRMS (ESI) calcd for C_40_H_66_ClFN_5_O_6_: 766.4680, found: 766.4685; ${\left[\alpha \right]}_{D}^{25}$-41.400 (MeOH, *c* = 0.50).

(*S*)-*N*-((3*R*,4*S*,5*S*)-1-((*S*)-2-((1*R*,2*R*)-3-(3-(4-Chloro-2-methoxyphenyl)azetidin-1-yl)-1-methoxy-2-methyl-3-oxopropyl)pyrrolidin-1-yl)-3-methoxy-5-methyl-1-oxoheptan-4-yl)-2-((*R*)-2-(dimethylamino)-3-methylbutanamido)-*N*,3-dimethylbutanamide (**1i**). Colorless oil, yield 45%; ^1^H-NMR (400 MHz, CDCl_3_) δ 7.15–7.10 (m, 1H), 7.01–6.92 (m, 1H), 6.87–6.84 (m, 1H), 4.88–4.73 (m, 3H), 4.56–4.50 (m, 1H), 4.45–4.41 (m, 1H), 4.37–4.06 (m, 4H), 3.99–3.91 (m, 2H), [3.83 (s) and 3.80 (s), total 3H], [3.44 (s) and 3.42 (s), total 3H], 3.39–3.29 (m, 5H), 3.14–3.01 (m, 4H), 2.82–2.76 (m, 1H), 2.63–2.24 (m, 7H), 2.31 (s, 3H), 2.29 (s, 3H), 2.18–1.77 (m, 9H), 1.37–1.13 (m, 10H), 1.05–0.79 (m, 9H); ^13^C-NMR (150 MHz, CDCl_3_) δ 174.4, 173.7, 173.5, 173.2, 171.2, 170.2, 157.9, 133.9, 133.6, 127.9, 127.5, 127.0, 120.6, 111.3, 86.2, 78.5, 78.0, 61.8, 59.4, 58.1, 57.8, 55.8, 53.8, 52.9, 52.6, 47.8, 46.6, 42.7, 39.3, 38.8, 37.4, 35.8, 32.9, 31.9, 28.8, 28.5, 26.0, 25.7, 24.9, 24.4, 23.6, 19.8, 19.4, 18.0, 17.8, 15.8, 13.9, 10.9; HRMS (ESI) calcd for C_41_H_69_ClN_5_O_7_: 778.4880, found: 778.4879; ${\left[\alpha \right]}_{D}^{25}$-33.400 (MeOH, *c* = 0.50).

### 3.2. Biological Evaluation Methodology

#### 3.2.1. Cancer Cell Proliferation Inhibition Assay

The following cell lines were used for the screening stage, obtained from American Type Culture Collection (ATCC, Manassas, VA, USA); HCT116 human colon cancer cells and A549 human lung carcinoma cell lines were cultured in RPMI 1640 medium supplemented with 10% FBS. Cell cultures were maintained in a humidified atmosphere of 5% CO_2_ at 37 °C. Cells were seeded at 2000 cells per well in 96-well plates in a volume of 200 μL per well. The test compounds were dissolved in DMSO and diluted with culture medium to different concentrations. After seeding for 24 h, the medium was removed, and 500 μL of the test compound solution was added in duplicates and incubation continued for 72 h at 37 °C in a humidified atmosphere containing 5% CO_2_. Control cells were treated with vehicle alone. During the last 4 h of incubation, the cells were exposed to tetrazolium dye (MTT) solution (5 mg/mL, 20 mL per well). The generated formazan crystals were dissolved in 100 mL of dimethyl sulfoxide (DMSO), and the absorbance was read spectrophotometrically at 570 nm using an enzyme-linked immunosorbent assay plate reader. The data was calculated using Graph Pad Prism version 5.0 (GraphPad Softwrae, La Jolla, CA, USA). The IC_50_s were fitted using a non-linear regression model with a sigmoidal dose response.

#### 3.2.2. *In Vivo* Efficacy Study

Pathogen-free, 4–6 week-old, female BALB/c athymic mice (Shanghai SCXK Laboratory Animal Technology Co. Ltd., Shanghai, China) were housed under sterile conditions. Human A549 xenograft was established in the right flanks of athymic mice according to the protocol of the National Cancer Institute. When the tumor reached a volume of 100 mm^3^, the mice were randomly assigned into control (*n* = 6 per group) and treatment groups (*n* = 6 per group). Control group were given lactate buffer, and treatment groups were iv administered with tested compounds. The size of tumor was measured individually on the indicated days. Tumor volume (V) was calculated as *V* = (length × width^2^)/2. The individual relative tumor volume (RTV) was calculated as follows: *RTV* = *V*_t_/*V*_0_, where *V*_t_ represented the last tumor size measurement and *V*_0_ represented the pre-dosing tumor size measurement. The animal experimental protocols were approved by the Animal Ethics Committee of School of Pharmacy, Fudan University and the mice were treated in accordance with international animal ethics guidelines.

## 4. Conclusions

In summary, we described the design and synthesis of nine TZT-1027 analogues based on the conformation restriction strategy. 3-Aryl-zetidines were used to replace the phenylethyl group at C-terminus. Two human cancer cell lines (A549, HCT116) were used to evaluate the potency of the synthesized compounds. Compound **1a** showed the strongest cytotoxic activities against A549 and HCT 116 cell lines (IC_50_ values were 2.2 nM and 2.1 nM, respectively). Compound **1a** could not achieve effective inhibition in A549 xenograft models at different dose levels. The poor solubility of **1a** limited a further exploration of *in vivo* activity at higher dosage. Our objective was to discover potent antitumor agents with novel scaffold. In this study, compound **1a** did not show any severe toxicity up to 5 mg/kg, showing a better safety potential than TZT-1027 (a dose of 4 mg/kg seemed to be toxic as reported).

## Figures and Tables

**Table 1:** IC_50_ values of compounds against A549 and HCT116 (MTT assay).

Compounds	A549 (nM ± SD) ^a^	HCT116(nM ± SD) ^a^
**1a**	2.2 ± 4.8	2.1 ± 0.4
**1b**	47.0 ± 9.9	2.3 ± 0.2
**1c**	35.0 ± 0.6	4.6 ± 0.7
**1d**	130.1 ± 48.3	25.5 ± 0.2
**1e**	19.5 ± 1.7	15.5 ± 1.8
**1f**	56.0 ± 3.3	3.7 ± 0.3
**1g**	41.5 ± 13.3	3.1 ± 0.8
**1h**	39.3 ± 2.4	8.3 ± 0.6
**1i**	8.7 ± 3.7	3.5 ± 0.9
**TZT-1027**	0.2 ± 0.06	0.3 ± 0.2
**Docetaxel**	23.5 ± 9.5	0.3 ± 0.1

^a^ The data were means from at least three independent experiments.

## Supplementary Materials

The following are available online at www.mdpi.com/1660-3397/14/5/85/s1, Table S1: Tumor Volume, Table S2: Relative Tumor Volume, Table S3: Tumor Growth Inhibition, Table S4: Solubility of **1a**.

## Abbreviations

The following abbreviations are used in this manuscript:

Dap	Dolaproine
Boc	*t*-Butyloxy carbonyl
HATU	2-(7-Aza-1*H*-benzotriazole-1-yl)-1,1,3,3-tetramethyluronium hexafluorophosphate
DIPEA	*N,N*-Diisopropylethylamine
MTT	3-(4,5-Dimethylthiazol-2-yl)-2,5-diphenyltetrazolium bromide
