# Supplementary material for: Novel Azetidine-Containing TZT-1027 Analogues as Antitumor Agents
Source: Mar Drugs. 2016 Apr 28;14(5):85. doi: 10.3390/md14050085 (PMC4882559; doi:10.3390/md14050085)
Supplement: Supplementary file 1 [file marinedrugs-14-00085-s001.pdf]

Supplementary Materials: Novel Azetidine-Containing TZT-1027 Analogues as Antitumor Agents

Qi Yan, Yujie Wang, Wei Zhang and Yingxia Li

Table S1. Tumor volume (mm^3, mean ± SD).

Days / Drug	1	4	7	10	13	16	19	22
Vechile	148.43 ± 76.26	220.42 ± 123.57	320.04 ± 150.33	412.44 ± 205.16	539.89 ± 264.96	725.23 ± 354.36	876.64 ± 389.87	992.21 ± 409.61
TZT-1027 2 mg/kg	147.31 ± 75.97	182.59 ± 71.09	196.64 ± 68.59	232.81 ± 85.13	275.81 ± 98.38	301.7 ± 95.66	346.59 ± 143.7	363.71 ± 153.23
1a 1 mg/kg	149.56 ± 73.72	185.88 ± 97.45	248.97 ± 144.16	341.75 ± 193.56	395.43 ± 199.2	530.82 ± 282.06	563.52 ± 321.07	662.64 ± 349.93
1a 2 mg/kg	16.16 ± 65.45	214.97 ± 104.56	287.02 ± 19.15	381.31 ± 124.24	508.66 ± 190.2	682.39 ± 265.79	813.25 ± 314.51	862.75 ± 456.75
1a 5 mg/kg	205.7 ± 95.63	266.39 ± 96.35	287.02 ± 83.11	380.17 ± 106.77	487.47 ± 110.65	554.26 ± 183.69	668.46 ± 210.57	789.59 ± 252.71

Table S2. Relative tumor volume (mean ± SD).

Days / Drug	1	4	7	10	13	16	19	22
Vechile	1	1.54 ± 0.53	2.39 ± 0.94	3 ± 1.06	3.94 ± 1.33	5.34 ± 1.99	6.47 ± 2.15	7.49 ± 2.8
TZT-1027 2 mg/kg	1	1.32 ± 0.4	1.48 ± 0.64	1.73 ± 0.78	2.09 ± 0.9	2.3 ± 0.9	2.6 ± 1.33	2.66 ± 1.11
1a 1 mg/kg	1	1.27 ± 0.37	1.65 ± 0.57	2.29 ± 0.76	2.69 ± 0.9	3.62 ± 1.05	3.86 ± 1.34	4.69 ± 1.74
1a 2 mg/kg	1	1.3 ± 0.22	1.8 ± 0.31	2.44 ± 0.46	3.22 ± 0.6	4.34 ± 0.97	5.15 ± 1.13	5.5 ± 2.15
1a 5 mg/kg	1	1.37 ± 0.34	1.53 ± 0.41	2.05 ± 0.62	2.7 ± 0.88	2.97 ± 0.91	3.51 ± 0.82	4.19 ± 1.41

Table S3. Tumor growth inhibition (%).

Days / Drug	1	4	7	10	13	16	19	22
TZT-1027 2 mg/kg	0.94 ± 0.04	4.99 ± 0.46	30.97 ± 0.38	1.73 ± 0.78	45.53 ± 0.2	53.94 ± 0.2	58.24 ± 0.2	61.94 ± 0.17
1a 1 mg/kg	−2.62 ± 0.17	10.68 ± 0.25	24.25 ± 0.23	2.29 ± 0.76	27.09 ± 0.15	28.02 ± 0.1	37.7 ± 0.13	34.79 ± 0.11
1a 2 mg/kg	1.97 ± 0.19	7.39 ± 0.15	11.09 ± 0.3	2.44 ± 0.46	8.85 ± 0.31	7.12 ± 0.27	11.14 ± 0.26	16.04 ± 0.35
1a 5 mg/kg	3.03 ± 0.23	−3.5 ± 0.36	17.97 ± 0.31	2.05 ± 0.62	17.78 ± 0.28	27.44 ± 0.3	33.4 ± 0.22	29.7 ± 0.3

Table S4. Solubility of **1a**.

	Solubility (Distilled Water, pH 7.4)
1a	346 μg/mL

Table S5. Apparent intrinsic clearance values of **1a** (Dose: 1 μM).

Compound	$T_{1/2}$ (min, Human)	Cl_{int} (mL/min/kg, Human)	$T_{1/2}$ (min, Rat)	Cl_{int} (mL/min/kg, Rat)	$T_{1/2}$ (min, Mouse)	Cl_{int} (mL/min/kg, Mouse)
**1a**	18.71	92.93	9.48	262.12	1.83	2981.16
**Ketanserin** [a]	43.86	39.63	15.62	159.00	13.54	402.92

[a] ketanserin is the reference compound.
